# A Case of Diabetic Ketoacidosis Presenting with Hypernatremia, Hyperosmolarity, and Altered Sensorium

**DOI:** 10.1155/2018/4806598

**Published:** 2018-07-16

**Authors:** Vinod Kumar, Sushant M. Nanavati, Gabriel Melki, Mira Upadhyaya, Raman Dhillon, Sandra Gibiezaite, Patrick Michael, Monisha Singhal

**Affiliations:** ^1^Department of Internal Medicine, St. Joseph's University Medical Center-New York Medical College, USA; ^2^Department of Family Medicine, St. Joseph's University Medical Center-New York Medical College, USA; ^3^Department of Endocrinology, St. Joseph's University Medical Center-New York Medical College, USA

## Abstract

Diabetic Ketoacidosis commonly presents with hyponatremia, but hypernatremia is a rare entity. We report a unique case of a 50-year-old woman admitted with altered sensorium with blood glucose 979 milligrams/deciliter, serum osmolarity 363 mOsm/kilograms, and serum sodium 144 milliequivalents/liter. Patient was given initial bolus of isotonic saline and continued on half isotonic saline for correction of hypernatremia along with insulin infusion therapy. Patient was successfully treated with intravenous fluids, insulin infusion, and the altered sensorium was resolved without any sequelae. This case illustrates a teaching point in the use of intravenous fluids for the treatment of Diabetic Ketoacidosis with hypernatremia.

## 1. Introduction

In the increasingly expanding population, Diabetes Mellitus accounts for 20-50% of new-onset diabetic patients in young adult population [1]. And Diabetic Ketoacidosis (DKA) continues to be the most severe medical emergency requiring admission to Intensive Care Unit (ICU). Here we discuss a unique case about a 44-years-old female presenting with hypernatremia in DKA secondary to severe hypotonic fluid loss and the management for this condition.

## 2. Case Presentation

A 50-years-old African female with medical history of hypertension, Diabetes Mellitus Type-2, and Major Depression Disorders presented with intractable vomiting and altered sensorium. About eight–ten hours prior to presentation, patient started to experience multiple episodes of nonbloody & nonbilious vomiting along with nausea leading to fatigue and altered sensorium requiring to be transported to hospital. Prior to initiation of the symptoms, she had suppressed appetite and skipped her dosage of Metformin 500 mg because of decreased oral intake and emesis. On presentation, patient was obtunded, responsive to pain, and poorly receptive to verbal stimuli. She had blood pressure of 123/81 mm Hg, respiratory rate of 25 breaths per minute, heart rate of 124 beats/minute, pulse oximetry of 97% on ambient air, and temperature of 97.6 Fahrenheit. On physical exam, she had mild distress, tachycardia, tenderness around epigastric area on deep palpation, and dehydration with poor skin turgor.

Due to state of presentation, computed tomography (CT) scan of the head showed no intracranial pathologies or cerebral edema presence. Venous blood gas showed pH 7.39, pCO_2_ 31 mm Hg, pO_2_ 52 mm Hg, HCO_3_ 18.8, sodium 148 mmol/L, potassium 3.5 mmol/L, glucose 750 mg/dl, and lactate 2.9 mmol/L. Initial biochemistry analysis showed serum sodium 144 meq/L, potassium 4.8 meq/L, chloride 98 meq/L, bicarbonate 14 meq/L, albumin 4.2 g/L, and serum glucose 979 mg/dl. Corrected sodium was calculated to be 158 meq/L, anion gap 32, delta gap: 2, and serum osmolality 363 mOsm/kg. Ketone bodies were strongly positive in the blood and urine.  Table 1 shows additional biochemical values appropriate to the time interval.

Patient had received initial fluid resuscitation and, later, she was admitted to ICU requiring administration of normal saline, initiation of intravenous insulin infusion, and electrolytes repletion. Serum glucose levels were appropriately improving with goal of 50-70 mg/dl per hours, though serum sodium continued initially to peak before the values started to decrease. Patient started to be alert, awake, and responsive to commands with tolerating oral diet and improvement from admission assessment. Serum sodium levels were gradually controlled within normal range over 72 hours within admission. Patient was eventually transferred to medical floor for optimization of diabetic medication and education prior to discharge without any further events.

## 3. Discussion

As hyperglycemia-induced osmotic fluid and osmotic diuresis occur between compartments, electrolyte disturbances are expected resultant in patients with DKA. In addition, they may present with complex metabolic interplay: Ketoacidosis or Ketoalkalosis [2].

Patients with DKA commonly present with hyponatremia on admission to the hospital. Uncontrolled plasma glucose causes increase in plasma tonicity, creating a driving force for the movement of water from intracellular space to extracellular space, which dilutes the extracellular concentration of sodium. In addition, secretion of vasopressin limits water loss via kidney, which all leads to hyponatremia [1, 3], while hypernatremia in DKA occurs from hypotonic renal losses, which is water excretion in excess of sodium and potassium, due to glycosuria-induced osmotic diuresis and inappropriate water replacement [3]. As per consensus statement guidelines, a correction factor of 1.6 mg per deciliter is added to the measured plasma sodium concentration for each 100 mg per deciliter of glucose above 100 mg per deciliter to account for the dilution effect of glucose. Corrected serum sodium provides a handy tool in monitoring and management during acute hyperglycemic crisis [1, 3]. In cases of high anion-gap acidosis, delta-delta or delta gap is employed to evaluate additional underlying metabolic acid-base disorder [1]. In our case, the anion gap was 32 mmol/liter and change (delta) in the concentration of bicarbonate ions was 10 mmol/liter with a calculated delta ratio of 2, which interprets anion-gap acidosis with concurrent metabolic alkalosis. In ketoacidosis, there is 1:1 correlation between the increase in the anion gap and the decrease in the concentration of bicarbonate, but the patient had severe hypernatremia that may have contributed to the anion gap causing increased delta gap [1].

There are numerous pediatric cases reported about hypernatremia in DKA secondary to new-onset Type 1 Diabetes Mellitus, carbonated carbohydrate beverages, and herbal product ingestion [4]. Most of these cases presented with the patient exhibiting altered sensorium, attributing to hypernatremia causing severe cellular dehydration with central nervous system (CNS) [4].

Some studies suggest altered sensorium determined by the levels of serum sodium, especially in non-ketotic hyperglycemia hyperosmolar syndrome [5, 6].

Management of hypernatremia in DKA constitutes of infusing 0.9% normal (isotonic) saline, at a rate of 15-20 milliliters per kilogram per hour or 1-1.5 liters during the first hour, to maintain effective plasma osmolality, in addition to supplementation of potassium [1, 7, 8]. Subsequently, fluids should be changed to 0.45% sodium chloride as the corrected sodium concentration may be in excess of 145 mmol/liter. One should take precautions to avoid risking cerebral edema, from overcorrecting acute hypernatremia, by decreasing plasma sodium concentration by 2 mol/liter/hour until plasma concentration is 145 mmol/liter [7].

In conclusion, our case provides clinicians with a refresher on the use of corrected sodium to guide the fluid therapy, altered sensorium driven by acute hypernatremia, and approach to the management of this condition.

## Figures and Tables

**Table 1:** 

Electrolytes	T: 0 (On admission)	T: 6	T: 12	T: 16	T: 20

Sodium (meq/L)	144	159	158	158	159

Potassium (meq/L)	4.8	3.4	3.9	4.1	3.9

Chloride (meq/L)	98	120	121	121	122

Bicarbonate (meq/L)	14	17	22	24	27

Calcium (mg/dl)	9.7	9.5	9.6	9.3	9.2

Phosphorus (mg/dl)	7.6	1.8	2.4	3	2.9

Magnesium (mg/dl)	3.1	2.4	2.1	2.2	1.9

Serum Glucose (mg/dl)	979	554	355	218	159

Albumin (G/L)	4.2	3.7	- - -	3.7	3.9

Creatinine (mg/dl)	2.43	1.57	1.39	1.47	1.37

Corrected Sodium (meq/L)	158	166	162	160	159

Anion Gap	32	22	16	13	10

Serum Osmolality (Calculated) mOsm/Kg	363	363	347	338	336

*∗*T: Time denoted in hours from admission.

## References

[1] Ingelfinger J. R., Palmer B. F., Clegg D. J. (2015). Electrolyte and Acid–Base Disturbances in Patients with Diabetes Mellitus. *The New England Journal of Medicine*.

[2] Kumar V., Nanavati S. M., Komal F. (2017). Ketoalkalosis: Masked Presentation of Diabetic Ketoacidosis With Literature Review. *Journal of Endocrinology and Metabolism*.

[3] Liamis G., Liberopoulos E., Barkas F., Elisaf M. (2014). Diabetes mellitus and electrolyte disorders. *World Journal of Clinical Cases*.

[4] Kim H. J., Kim D. H., Jun Y. H., Lee J. E. (2014). A rare diabetes ketoacidosis in combined severe hypernatremic hyperosmolarity in a new-onset Asian adolescent with type I diabetes. *BMJ Case Reports*.

[5] Liamis G., Tsimihodimos V., Doumas M., Spyrou A., Bairaktari E., Elisaf M. (2008). Clinical and laboratory characteristics of hypernatraemia in an internal medicine clinic. *Nephrology Dialysis Transplantation *.

[6] Popli S., Leehey D. J., Daugirdas J. T. (1990). Asymptomatic, nonketotic, severe hyperglycemia with hyponatremia. *JAMA Internal Medicine*.

[7] Ingelfinger J. R., Sterns R. H. (2015). Disorders of plasma sodium—causes, consequences, and correction. *The New England Journal of Medicine*.

[8] Kitabchi A. E., Umpierrez G. E., Miles J. M., Fisher J. N. (2009). Hyperglycemic crises in adult patients with diabetes. *Diabetes Care*.

